# Prehospital and Posthospital Fall Injuries in Older US Adults

**DOI:** 10.1001/jamanetworkopen.2020.13243

**Published:** 2020-08-19

**Authors:** Geoffrey J. Hoffman, Mary E. Tinetti, Jinkyung Ha, Neil B. Alexander, Lillian C. Min

**Affiliations:** 1 Department of Systems, Populations and Leadership, University of Michigan School of Nursing, Ann Arbor; 2 Institute for Healthcare Policy and Innovation, University of Michigan, Ann Arbor; 3 Department of Medicine (Geriatrics), Yale University, New Haven, Connecticut; 4 School of Public Health, Yale University, New Haven, Connecticut; 5 Division of Geriatric and Palliative Medicine, Department of Internal Medicine, University of Michigan, Ann Arbor; 6 Geriatric Research Education Clinical Center, VA Medical Center, Ann Arbor, Michigan; 7 Veterans Affairs Center for Clinical Management and Research, VA Medical Center, Ann Arbor, Michigan; 8 Institute for Social Research, University of Michigan, Ann Arbor

## Abstract

**Importance:**

To date, measurement and treatment of older adult fall injury has been siloed within specific care settings, such as a hospital or within a nursing home or community. Little is known about changes in fall risk across care settings. Understanding the occurrence of falls across settings has implications for measuring and incentivizing high-value care across care settings.

**Objective:**

To estimate the risk of older adult fall injury within and across discrete periods during a 12-month care episode anchored by an acute hospitalization.

**Design, Setting, and Participants:**

This cohort study is a longitudinal analysis of 12-month periods that include an anchor hospital stay using national data from 2006 to 2014. Participants included older (aged ≥65 years) Medicare fee-for-service beneficiaries from the Health and Retirement Study. Weekly fall injury rates were computed for 4 periods compared with the anchor hospitalization: at baseline (1-6 months before hospitalization), just before (<1 month before hospitalization), just after (<1 month after hospitalization), and at follow-up (1-6 months after hospitalization). Piecewise logistic regression models estimated weekly marginal risk of fall injury within each period, adjusting for sociodemographic and health characteristics. Fall injury risks for high-risk beneficiaries with a fall injury during the anchor hospitalization were also estimated. Data analysis was performed from November 2019 to April 2020.

**Main Outcomes and Measures:**

Fall injuries.

**Results:**

In total, 10 106 anchor hospitalizations for 4101 beneficiaries (mean [SD] age, 77.1 [7.6] years; 5912 hospitalizations among women [58.5%]) were identified. The overall fall injury risk was 0.77%. In adjusted models, marginal increases in weekly fall injury risk just before hospitalization (0.27 percentage points [95% CI, 0.22 to 0.33 percentage points], or 30.0%; *P* < .001) were 4 times greater than decreases just after hospitalization (−0.18 percentage points [95% CI, −0.23 to −0.13 percentage points], or −9.2%; *P* < .001)]. A greater risk differential before and after hospitalization was observed for patients with an inpatient fall injury (1.89 percentage points [95% CI, 1.37 to 2.40], or 309.8%; *P* < .001; vs −0.39 percentage points [95% CI, −0.73 to −0.04], or −11.6%; *P* = .03).

**Conclusions and Relevance:**

An episode-based assessment of fall injury illustrates substantial variability in period-specific risks over an extended period including an anchor hospitalization. Risk transitions between periods include sizable increases just before hospitalization that do not fully subside after hospital discharge. Financial incentives to coordinate hospital and posthospital care for patients at risk for fall injury are needed. These could include bundled payments for fall injury episodes that incentivize coordination across settings.

## Key Points

**Question:**

What are older adults’ risks of fall injuries in the periods surrounding hospitalization?

**Findings:**

This retrospective cohort study using national survey and linked Medicare data observed spikes in older adult fall injury risk in the periods just before and after hospitalization. Risk increases were particularly pronounced for those who experienced an inpatient fall injury.

**Meaning:**

These findings suggest that efforts to improve coordination of fall injury risk during care transitions into and out of the hospital are needed.

## Teaser

This cohort study estimates the risk of older adult fall injury within and across discrete periods during a 12-month care episode anchored by an acute hospitalization.

## Introduction

Falls are a persistent and costly public health threat.^1,2^ Absent prevention, fall injury risks can compound,^3,4,5,6,7^ creating long-term disability plus increased need for acute^2,8^ and postacute needs,^9,10^ as well as hospital readmissions.^11^ Organized efforts to prevent and treat falls in older adult typically occur discretely within communities, hospitals, or skilled nursing facilities,^12,13,14,15,16^ an approach resulting in limited coordination or oversight across care settings.^11,17,18,19^ Even as Medicare increasingly incentivizes care coordination among clinicians with new payment models, fall injuries continue to be addressed and measured primarily within single settings.

However, little is known about changes in fall injury risk across care episodes, particularly those involving hospitalizations. Fall injury risk may be exacerbated during and after a hospital stay when patients are immobilized, sedated, experience adverse effects of medications, or do not receive adequate fall-related patient education and rehabilitation at discharge.^11,18,19,20,21,22^ On arrival at a hospital, patients may have exacerbated fall injury risk due to functional, cognitive, or health issues,^9^ given that falls can be markers of underlying disablement or chronic illness.^23,24,25^ If preexisting risks are already high but then increase during hospitalization, risks for fall injuries may be greatest among recently discharged patients.

Prior research^9^ using a smaller, hospital-based sample observed a 4-fold decrease in falls from 2 weeks compared with 3 months after a hospital discharge, but was unable to determine whether heightened risk just after hospitalization reflected hospital care, preexisting risk, or both, because it examined data from after the hospital stay only. We sought to understand changes in fall injury risk across a care episode anchored by an acute hospitalization, using survey and linked claims data from inpatient, outpatient, and postacute care settings. We measured changes in prehospitalization and posthospitalization risks of fall injury over a 12-month period, including for patients who experienced an inpatient fall injury. In the context of changing Medicare payment methods, our work can extend our understanding of fall injury risk beyond setting-specific risks to comprehensively illustrate changes in risk across complete care episodes.

## Methods

### Data Sources

This study was approved by the institutional review board at the University of Michigan, which deemed the study to pose no more than minimal risk to participants; thus, informed consent was not sought. The study follows the Strengthening the Reporting of Observational Studies in Epidemiology (STROBE) reporting guideline.

We used linked Medicare claims and the Health and Retirement Study (HRS), a biennial survey assessing health and functioning of adults aged 51 years and older. For its bundled payment policy, Medicare uses care episodes anchored by a hospitalization. In light of this episodic approach, we used Medicare hospital, skilled nursing facility, outpatient, and physician visit administrative files from 2007 to 2014 to identify anchor hospitalizations and 180-day observation periods surrounding hospitalization. We excluded admissions with discharge dates after April 10, 2014 (allowing for a 6-month follow-up), to avoid classification bias associated with the introduction of *International Statistical Classification of Diseases and Related Health Problems, Tenth Revision* on October 1, 2014. To adjust for beneficiary risk, we merged administrative data with patient 2006 to 2012 survey data from the HRS.

### Study Population

The cohort included all 12-month observation periods (ie, periods including 6 months before and 6 months after the hospitalization). The anchor hospitalization did not have to include a fall injury. We identified 10 106 hospitalizations for 4101 unique older (aged ≥65 years) Medicare beneficiaries alive and with fee-for-service coverage for the approximately 12-month study period. See cohort derivation in eTable 1 in the Supplement.

### Primary Outcome

Our primary outcome was a fall-related injury that captured fractures, joint dislocations, strains, and contusions, identified with an algorithm we recently developed and validated^26^ using *International Classification of Diseases, Ninth Revision* diagnoses, procedure, and external cause-of-injury codes from all administrative sources. If health care for the same fall injury was provided within 30 days, those encounters were considered as a single fall injury episode (aggressive approach) to avoid overcounting multiple treatments for a single injury. In a separate conservative approach, we classified injury diagnoses involving the same area of the body (eg, a left leg contusion followed by fracture of the right ankle), within 30 days of one another, as a single fall injury episode, thus potentially classifying some new injuries as nonincident.

### Covariates

We controlled for sociodemographic and health characteristics associated with falls and hospitalization and fall risk^27^: a continuous measure of age, sex, race/ethnicity (African-American, Hispanic, non-Hispanic White, or other), educational level (less than high school, some college, college, more than college), and separate indicator variables for each of 7 chronic conditions (diabetes, cancer, lung disease, heart problems, stroke, psychological problems, and arthritis), and cognitive impairment measured using the Telephone Interview of Cognitive Status and proxy responses.^28^ A binary frailty indicator was constructed using 4 functional domains (physical, nutritive, cognitive, and sensory), where an individual was considered frail if she or he had difficulty in 2 or more domains.^29^ More details on variables are available in eTable 2 in the Supplement.

These measures were extracted from the HRS survey wave closest to but preceding the start of the observation period (ie, the date 6 months before the anchor hospitalization). The mean (SD) difference from the interview to the study start was 386 (218.1) days (eFigure in the Supplement).

### Statistical Analysis

We described the sample and assessed the hospital diagnosis using diagnosis-related groups and computed weekly unadjusted fall injury rates. Next, we defined 4 periods: baseline (1-6 months before the hospitalization), just before (<1 month) and after (<1 month) hospitalization, and follow-up (1-6 months after hospitalization). The period of hospitalization was not included; therefore, the month-long period just following hospitalization was measured from the date of discharge plus 30 days. In the Figure, the weeks before and after hospitalization are referenced as weeks −1 and week 1, respectively, and the hospitalization period (which could last >1 week) as time 0; therefore, 4 weeks before and after hospitalization were referenced as weeks −5 and week 5 in the Figure.

**Figure.**
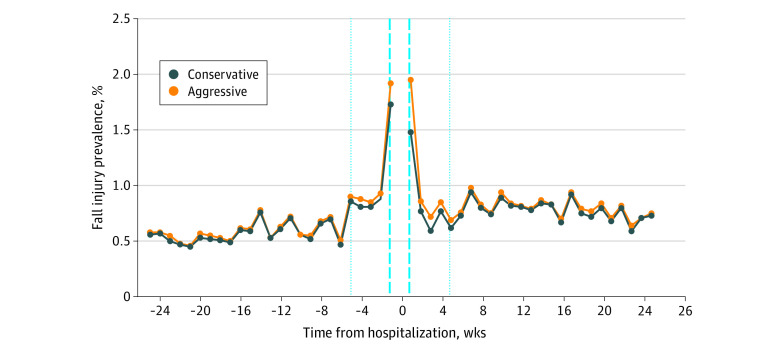
Predicted Probabilities of Fall Injury Among Older Adults in the Period Surrounding a Hospitalization Predicted fall injury prevalence from an unadjusted piecewise logistic regression model with points of interest indicated, at −25 weeks (6 months before hospitalization), −5 weeks (1 month before hospitalization), 5 weeks (1 month after hospitalization), and 25 weeks (6 months after hospitalization). The conservative injury algorithm classifies fall injury diagnoses as part of an earlier fall injury episode if the diagnosis involves the same body part (eg, lower extremities, upper extremities, neck or trunk, and head injury). The aggressive injury algorithm classifies fall injury diagnoses as part of an earlier fall injury episode only where the diagnosis code has the same leading 3 digits; in all other cases, a fall injury diagnosis is considered as a new fall injury. Fall injuries that resulted in a hospitalization (ie, a fall injury treated in an outpatient setting that then resulted in a hospitalization) were not included in computing the probability of a fall injury in the month before hospitalization (meaning increases in fall injuries just before hospitalization are not a direct result of prehospital fall injuries). Dashed blue lines denote the period of hospitalization. The dotted blue line on the left denotes the beginning of the prehospitalization period, and the dotted blue line on the right denotes the end of the posthospitalization period.

We estimated piecewise logistic regression models that fit multiple models to the data (for the 4 periods of interest) by implicitly recognizing different functions that can be fit to different ranges of data.^30^ The breakpoints separating the 4 periods are where the slopes of the logistic regressions change; that is, different coefficients are estimated for each of the 4 periods. The breakpoints were prespecified to reflect policy interest in the 30-day postdischarge period and to establish longer baseline and follow-up periods that allow us to assess whether temporal changes occur in fall injury patterns. An additional description of piecewise logistic regression provided in eTable 3 in the Supplement. For ease of interpretation, we computed marginal effects (using the *margins* command in Stata statistical software). These can be interpreted as the change in the adjusted probability of a fall injury (after accounting for all model covariates) for an additional week spent in a given time period (ie, weekly percentage-point change in risk). Using the initial risk from each period (ie, during the first week of a period), the marginal effects were used to calculate the average weekly percentage change in risk for each period.

A first model included all model covariates plus a year dummy variable to account for secular trends. Patients who experience inpatient fall injuries may have unique fall injury risks; therefore, we examined whether the risks of fall injuries varied across care episodes according to whether patients experienced a fall injury during a hospital stay (327 observations for fall injuries not present at admission). Because 2259 individuals had multiple hospitalizations, we used cluster-robust SEs. All *P* values were 2-sided, and *P < *.05 was set as the threshold for significance. Inclusion of quadratic terms for the time variables for each period were explored, but results were not substantially changed (ie, the probabilities of fall injury risk were fairly constant within each period). The main model was re-estimated using the conservative fall injury episode approach and produced similar results (eTables 4 and eTable 5 in the Supplement).

We performed several sensitivity analyses. First, we examined whether period-specific risks differed according to the anatomic body part (head, neck or trunk, upper body, or lower body) injured in prehospitalization falls.^26^ Second, we re-estimated the main model using only individuals for whom the time between the HRS survey date and start of the study period was equal to 1 year or less. Third, we dropped the first hospitalization for individuals with multiple hospitalizations. Fourth, rather than a 30-day period without fall diagnoses to define distinct fall injuries, we used a 180-day period.

Statistical analysis was performed with SAS statistical software version 9.4 (SAS Institute) and Stata MP statistical software version 15 (StataCorp). Analysis was performed from November 2019 to April 2020.

## Results

### Patient Characteristics

We identified 10 106 eligible hospitalizations for 4101 unique older Medicare beneficiaries. As shown in Table 1, the mean (SD) age of respondents was 77.1 (7.6) years, with 5912 hospitalizations among women (58.5%) and 7630 hospitalizations (75.5%) among non-Hispanic White patients. At the index hospitalization, approximately 2600 patients had circulatory system diseases, and approximately 1000 patients each had diagnoses of respiratory system, digestive system, and connective tissue diseases. Metabolic issues (168 patients), depression (158 patients), postoperative infection (62 patients), and pneumonia (48 patients) were also diagnosed. Fall rates per 1000 patient-days are provided in eTable 6 in the Supplement.

**Table 1.** Characteristics of Older (Aged ≥65 y) Individuals, by Cognitive Impairment and Frailty Status, 2008-2014

Characteristic	All hospitalizations (N = 10 106), No. (%)^a^	First hospitalizations only (n = 4101), No. (%)^b^
Age, mean (SD), y	77.1 (7.6)	76.3 (7.7)
Female	5912 (58.5)	2411 (58.8)
Education		
No degree	2840 (28.1)	1050 (25.6)
General equivalency diploma or high school	5323 (52.7)	2217 (54.1)
College	1271 (12.6)	537 (13.1)
Master’s or professional degree	672 (6.7)	297 (7.2)
Race/ethnicity		
Non-Hispanic White	7630 (75.5)	3220 (78.5)
African-American	1531 (15.2)	511 (12.5)
Hispanic	759 (7.5)	299 (7.3)
Other	186 (1.8)	71 (1.7)
Frail	3537 (35.0)	1177 (28.7)
Cognitively impaired	781 (7.7)	238 (5.8)
Chronic condition		
Diabetes	3335 (33.0)	1147 (28.0)
Cancer	2299 (22.8)	823 (20.1)
Lung disease	1933 (19.1)	595 (14.5)
Heart problems	4859 (48.1)	1649 (40.2)
Stroke	1604 (15.9)	478 (11.7)
Psychological problems	2276 (22.5)	784 (19.1)
Arthritis	7887 (78.0)	3072 (74.9)

^a^ All hospitalizations indicates all anchor hospitalizations in the main analysis, including multiple hospitalizations from individuals.

^b^ First hospitalizations indicates respondents’ first hospitalizations (ie, excluding hospitalizations after the first 1 for individuals with multiple hospitalizations).

#### Unadjusted Fall-Related Injury Prevalence

As shown in the Figure, weekly probabilities of fall-related injuries assessed using the claims data were 0.58% at the beginning of the baseline period, nearly doubled (0.90%) 1 month before hospitalization, and then doubled again (1.92%) 1 week before hospitalization. The risks remained high (1.95%) 1 week after hospitalization and then decreased (to 0.69%) by 1 month after hospitalization, before slightly increasing (0.75%) at the end of the follow-up period.

For those with an inpatient fall, variation was greater: 1.22% at baseline, 0.61% 1 month before hospitalization, 1.53% 1 month after hospitalization, and 1.83% at follow-up. The probabilities were higher in the week just before (9.79%) and just after (7.34%) hospitalization.

#### Risks of a Fall Injury Across a 12-month Episode

We estimated the change in the risk of a fall injury for each period (Table 2). The overall fall injury risk was 0.77%. At baseline, the risk of a fall injury increased by 0.02 percentage points (95% CI, 0.01 to 0.03 percentage points), or 3.4%, each week (*P* = .01). Just before hospitalization, fall injury risk increased by 0.27 percentage points (95% CI, 0.22 to 0.33 percentage points), or 30.0% (*P* < .001) each week, or 120.0% during the entire 4-week period. Just after hospitalization, the risk decreased (−0.18 percentage points [95% CI, −0.23 to −0.13 percentage points], or −9.2% each week, or −36.8% in total) but not as much as the prehospital increase. At follow-up, there was no change in the risk of a fall injury.

**Table 2.** Marginal Effects for a Fall Injury for Older (Aged ≥65 y) Adults During 4 Periods Before and After a Hospitalization Overall and for Inpatients Who Experienced a Fall

Period^a^	Overall (n = 10 106)			Inpatients who experienced a fall (n = 327)^b^		
	Marginal effect, percentage points (95% CI)^c^	Initial risk, %	Change, %	Marginal effect, percentage point (95% CI)^c^	Initial risk, %	Change, %
Baseline	0.02 (0.01 to 0.03)^d^	0.58	3.4	−0.06 (−0.14 to 0.02)	1.22	−4.9
Hospitalization						
Before	0.27 (0.22 to 0.33)^d^	0.90	30.0	1.89 (1.37 to 2.40)^d^	0.61	309.8
After	−0.18 (−0.23 to −0.13)^d^	1.95	−9.2	−0.39 (−0.73 to −0.04)^d^	3.36	−11.6
Follow-up	0.00 (−0.01 to 0.00)	0.69	0.0	−0.04 (−0.08 to 0.00)^d^	0.92	−4.3

^a^ Baseline refers to 6 to 1 month before hospitalization; before hospitalization refers to less than 1 month before hospitalization; after hospitalization refers to from discharge to 1 month following discharge; follow-up refers to 1 to 6 months following discharge.

^b^ Refers to individuals who experienced a fall during the hospitalization (ie, had a fall injury that was not present on admission).

^c^ A marginal effect of 0.27 in the before-hospitalization period can be interpreted as a weekly increase of 0.27 percentage points in the 4 weeks before hospitalization. Because the risk at the beginning of this period was 0.90 (a 0.90% probability of a fall injury), the weekly mean increase is 0.27 / 0.90 = 30.0%.

^d^ *P* < .05.

We also estimated changes in risk, by period, for only those 327 respondents who experienced an inpatient fall injury. The prehospital changes were much larger for this high-risk subsample. For the baseline period, fall injury risk was unchanged. Just before hospitalization, it increased sharply by 3-fold per week (309.8%, or 1.89 percentage points; 95% CI, 1.37 to 2.40 percentage points; *P* < .001). Just after hospitalization, risk decreased only by 11.6% per week (−0.39 percentage points; 95% CI, −0.73 to −0.04 percentage points; *P* = .03) and at follow-up, risk decreased by 4.3% each week.

### Sensitivity Analyses

In a first sensitivity analysis, results were similar for patients with a head, neck or trunk, or upper or lower body injury, with risks increasing both at baseline and just before, but not changing in either of the periods after hospitalization (eTable 7 in the Supplement). Results from the main model were robust to the 3 additional sensitivity analyses (eTable 8 in the Supplement).

## Discussion

In this study of 12-month fall injury risks for older adults with an anchor hospitalization, we report 2 main findings. First, risks were disproportionately large in the month just before hospitalization. Although risks decreased in the month after discharge, the increases in fall injury risk before hospitalization (30.0% each week, or >120.0% in total) were 3 times greater than the subsequent risk decreases just after hospitalization (−9.2% each week, or −36.8% in total), although risk sizably subsided later on. Second, changes in fall injury risk across care settings were substantial, most notably for patients who experienced an inpatient fall injury. For those patients, fall injury risk increased by 309.8% each week in the month just before hospitalization and did not subside as much after hospitalization. In all, our findings suggest opportunities to systematically address fall injury risks, particularly for high-risk older adults, through posthospital transitional fall injury prevention and possibly through the introduction of incentives to coordinate care for patients throughout a care episode, across care settings.

A 2000 study^9^ reported high posthospital fall incidence, with fall rates 4 times greater just after discharge compared with 10 weeks after hospital discharge for 311 patients ages 65 and older. Our work confirms that risk of fall injury is high just after discharge but further observes sizeable increases in prehospital fall injury risk that are not completely stabilized, or eliminated, after the hospitalization. In all, episodic fall injury risk appears to be a product of both prehospital illness and functional challenges as well as inpatient fall-related care quality issues, such as reduced inpatient mobility and activity, iatrogenic complications, poor nutrition and rest, and limited clinician education and referrals regarding fall prevention interventions.^11,18,19,22,31,32^ It also likely reflects gaps in posthospital rehabilitation and therapeutic support.

Considerable work shows that transitions to disability or death are common after falls and critical illness requiring hospitalization.^3,4,5,6,7^ Our work similarly observed risk transitions, with substantial risk increases preceding hospitalization for inpatients who experienced a fall, highlighting the importance of early promotion of physical function through community and home-based prevention efforts.^16^ Enlargement of these interventions to include prehospital transitional care handoffs (between clinicians in community settings, as well as between primary care clinicians and hospital clinicians upon admission) could benefit patients, particularly vulnerable ones, and improve on siloed efforts occurring in discrete care settings.^14,16,33,34,35,36,37^

Improvements in such handoffs may help shift from a point-in-time to an episodic fall injury prevention approach, to identify and address risks before, during, and after hospitalization. Such an approach could address current gaps in multidisciplinary care coordination for at-risk individuals aiming to access the right clinician at the right time,^38^ whereas presently needed services are often dislocated; a home safety check or rehabilitative care may not be obtained unless an individual recently experienced a hospitalization, even though posthospital fall risks extend well beyond a hospitalization, or a fall prevention intervention referral may not be provided at the hospital discharge if hospital clinicians are unaware of patients’ fall history.

These findings have implications for how current policy incentivizes prevention of fall injuries across the care continuum. Under current policies, prevention is segmented by care setting, in ways that may augment risk elsewhere. Medicare’s never-events policy penalizing hospitals for inpatient falls may redistribute risk into the posthospital setting by discouraging inpatient mobility.^17^ Medicare’s readmissions penalties may restrict attention to the 30-day period after discharge, ignoring continuing risks in the longer-term period after hospitalization.

Moreover, fall injuries are broadly excluded from Medicare’s bundled payments policy, which aligns incentives of clinicians across care settings.^39,40^ In Medicare’s program, episodes last 90 days following the anchor hospitalization or procedure.

Under this episode-based framework, there are incentives for clinicians from different care settings to work with one another to coordinate and improve care. With these incentives, hospital clinicians might better leverage multidisciplinary staff at the time of admission to target resources for inpatient fall reduction both during the hospitalization as well as during transitional fall prevention efforts on discharge to reduce posthospital falls.^17,20,41^ This could also include partnerships and coordination with outpatient and community clinicians to ensure up-to-date medication checks, enrollment in prevention interventions, and home safety visits, much in the way that hospitals have addressed patient readmission risks.^42,43^ Bundled payments might remove incentives hospitals have for immobilizing patients to avoid penalties, given that immobilization of patients may hamper postacute functional rehabilitation, thus increasing fall risk.

Given our results, we believe that better community-based prehospital fall prevention should also yield financial benefits to hospitals and posthospital care clinicians, because bundled payments align incentives between hospitals and community-based organizations. Shared incentives under Medicare’s Hospital Readmissions Reduction Program resulted not only in reduced readmissions but also reduced overall hospitalizations.^43^ In the same way, partnerships between these clinicians could strengthen fall prevention; for instance, by connecting with patients’ primary care clinicians to understand prehospital fall history, hospital clinicians might better plan for posthospital episodic risk reduction that reduces future fall risks.

### Limitations

This study had several limitations. First, our findings cannot be used to infer that someone with a fall will be hospitalized, given that we conditioned on hospitalization and because hospitalized patients are not a representative population. Second, we did not capture falls that did not require health care, but our validated algorithm was designed to maximize capture of both injurious falls and fall-related health care in claims data.^26^

Third, some fall injuries could result in hospitalization, which could have increased the observed incidence of fall injuries in the immediate prehospitalization period. If so, the sharply increased prehospital risk could have been colinear with the hospitalization. We addressed this by excluding from the prehospital period any fall injuries for patients who subsequently, within the following 30 days, had a hospital fall injury diagnosis. For instance, if a patient had a fall injury treated in an outpatient setting and was sent home but was subsequently admitted to the hospital for the same diagnosis, we did not count that diagnosis as a prehospital fall injury. Fourth, the results may have been affected by loss-to-follow up bias: if individuals at greater risk for fall injuries died after hospitalization, we may have understated posthospital injury risk. Fifth, our study design, which included multiple and, in some cases, overlapping anchor hospitalizations for individuals, sizeable time lags between the anchor hospitalization and HRS survey in which risk factors were measured, and the 30-day fall injury episode construction, could have influenced our findings. However, our main findings were robust, with similar prehospital spikes and less steep posthospital decreases, compared with using alternative approaches.

## Conclusions

These findings suggest that fall injuries increase 3-fold just before a hospital stay, with risks remaining high just after hospitalization before somewhat subsiding. Current financial incentives under Medicare may discourage collaboration of clinicians across settings, muting potential prevention efficacy. To address this, Medicare should consider including fall injuries as a condition assessed under the bundled payments program, which could incentivize hospitals and community-based clinicians and organizations to assess and address fall injury risk across the care continuum.

## Notes

Supplement. **eTable 1.** Cohort Derivation **eTable 2. **Further Description of Model Covariates **eFigure.** Illustration of the Time Between Health and Retirement Survey Interview and Start of the Study Period (6 Months Prior to Anchor Hospitalization) **eTable 3.** Description of Piecewise Logistic Regression Model **eTable 4.** Odds Ratios and Marginal Effects Using Conservative Approach **eTable 5.** Odds Ratios and Marginal Effects Using Aggressive Approach **eTable 6.** Fall Injury Rates (per 1,000 Patient Days) of Older (≥65) Individuals, Relative to Hospitalization, 2008-2014 (*n*=10,106) **eTable 7.** Marginal Effects (MEs), and 95% Confidence Intervals (CI) for a Fall Injury for Older (≥65) Adults with a Pre-Hospital (Month Before Hospitalization) Fall During Four Time Periods Before and After a Hospitalization by Injured Body Part (*n*=433) **eTable 8.** Sensitivity Analyses Indicating Marginal Effects and 95% Confidence Intervals (CI) for a Fall Injury for Older (≥65) Adults during Four Time Periods Before and After a Hospitalization
