# Supplementary material for: Prehospital and Posthospital Fall Injuries in Older US Adults
Source: JAMA Netw Open. 2020 Aug 19;3(8):e2013243. doi: 10.1001/jamanetworkopen.2020.13243 (PMC7439104; doi:10.1001/jamanetworkopen.2020.13243)
Supplement: Supplement. — eTable 1. Cohort Derivation eTable 2. Further Description of Model Covariates eFigure. Illustration of the Time Between Health and Retirement Survey Interview and Start of the Study Period (6 Months Prior to Anchor Hospitalization) eTable 3. Description of Piecewise Logistic Regression Model eTable 4. Odds Ratios and Marginal Effects Using Conservative Approach eTable 5. Odds Ratios and Marginal Effects Using Aggressive Approach eTable 6. Fall Injury Rates (per 1,000 Patient Days) of Older (≥65) Individuals, Relative to Hospitalization, 2008-2014 (n=10,106) eTable 7. Marginal Effects (MEs), and 95% Confidence Intervals (CI) for a Fall Injury for Older (≥65) Adults with a Pre-Hospital (Month Before Hospitalization) Fall During Four Time Periods Before and After a Hospitalization by Injured Body Part (n=433) eTable 8. Sensitivity Analyses Indicating Marginal Effects and 95% Confidence Intervals (CI) for a Fall Injury for Older (≥65) Adults during Four Time Periods Before and After a Hospitalization [file jamanetwopen-3-e2013243-s001.pdf]

Supplementary Online Content

Hoffman GJ, Tinetti ME, Ha J, Alexander NB, Min LC. Prehospital and posthospital fall injuries in older US adults. *JAMA Netw Open*. 2020;3(8):e2013243. doi:10.1001/jamanetworkopen.2020.13243

eTable 1. Cohort Derivation
eTable 2. Further Description of Model Covariates
eFigure. Illustration of the Time Between Health and Retirement Survey Interview and Start of the Study Period (6 Months Prior to Anchor Hospitalization)
eTable 3. Description of Piecewise Logistic Regression Model
eTable 4. Odds Ratios and Marginal Effects Using Conservative Approach
eTable 5. Odds Ratios and Marginal Effects Using Aggressive Approach
eTable 6. Fall Injury Rates (per 1,000 Patient Days) of Older (≥65) Individuals, Relative to Hospitalization, 2008-2014 (*n*=10,106)
eTable 7. Marginal Effects (MEs), and 95% Confidence Intervals (CI) for a Fall Injury for Older (≥65) Adults with a Pre-Hospital (Month Before Hospitalization) Fall During Four Time Periods Before and After a Hospitalization by Injured Body Part (*n*=433)
eTable 8. Sensitivity Analyses Indicating Marginal Effects and 95% Confidence Intervals (CI) for a Fall Injury for Older (≥65) Adults during Four Time Periods Before and After a Hospitalization

This supplementary material has been provided by the authors to give readers additional information about their work.

© 2020 Hoffman GJ et al. *JAMA Network Open.*

eTable 1. Cohort Derivation

	No. of patients	No. of hospitalizations
Medicare beneficiaries ages 65 and older	6,122	17,491
Part A/B eligible	5,212	13,541
Discharge date < 9/1/2014	4,999	12,607
Link with HRS core interviews	4,294	10,614
No missing covariates	4,101	10,106

© 2020 Hoffman GJ et al. *JAMA Network Open.*

eTable 2. Further Description of Model Covariates

Assessing Cognitive Impairment	To identify cognitive impairment, we followed existing practice by using the Telephone Interview of Cognitive Status (TICS), an alternative to the Mini-Mental State Examination.[46,47] TICS scores range from 0-35, with higher scores indicating better functioning. Respondents with scores of ≤8 were considered impaired.[28] Because not all respondents were able to complete the TICS, we followed precedent by using information reported from proxy respondents regarding respondent's cognitive status, which were assessments of the respondent's memory (0-4); limitations in instrumental activities of daily living (0-5); and the respondent's difficulty in completing the interview because of cognitive impairment (0-2).[28] This resulted in a scale with a range of 0-11, with higher scores representing worse cognition; individuals whose proxies reported scores of ≥6 were additionally considered cognitively impaired.
Race/Ethnicity	The HRS offers seven response categories for race plus an "other" category; the authors collapsed categories with small numbers (American Indian, Alaska Native, Asian, Native Hawaiian, Pacific Islander, other) into a single "Other" category. These were collapsed, due to small cell sizes, into four categories: African-American, Hispanic, non-Hispanic White, and Other.
Frailty	The frailty measure is based on an indicator developed by Cigolle et al. (2009)[29] using variables from the Health and Retirement Study and includes 4 domains: physical, nutritive, cognitive, and sensory. Individuals with difficulties in two or more domains were considered frail. Problems with physical functioning were indicated when dizziness was a persistent problem, if the respondent had two or more falls in the prior 2 years, or difficulty lifting 10 pounds. Problems with nutritive functioning were indicated if the respondent had a 10% or greater weight loss in the prior 2 years or a BMI of <18.5 kg/m^2. Problems with cognitive functioning were indicated using self and proxy reports, as indicated above. Problems with sensory functioning were indicated by fair or poor vision or hearing despite the use of corrective lenses or hearing aides. See Cigolle et al. for more.[29]

© 2020 Hoffman GJ et al. *JAMA Network Open.*

eFigure. Illustration of the Time Between Health and Retirement Survey Interview and Start of the Study Period (6 Months Prior to Anchor Hospitalization)

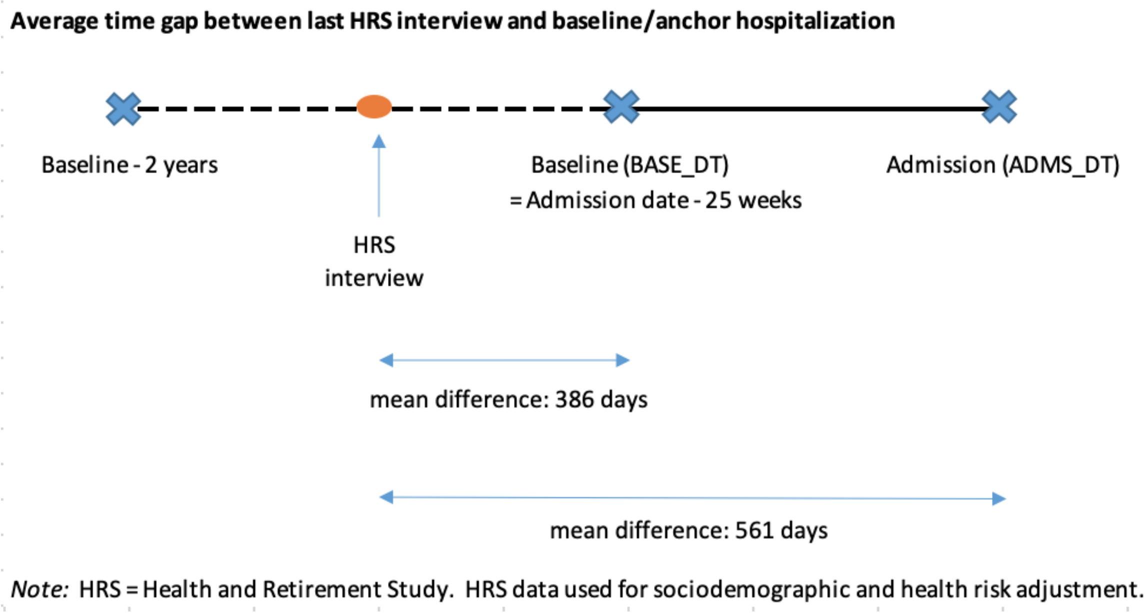

© 2020 Hoffman GJ et al. *JAMA Network Open.*

eTable 3. Description of Piecewise Logistic Regression Model

For the time trend, in total we have 4 variables, and they are all continuous:

Time 1 variable for periods 1 and 2,
Time 2 variable for period 2,
Time 3 variable for periods 3 and 4,
Time 4 variable for period 4

Time 1 and 3 variables are baseline variables for pre and post hospitalization. They are not connected because of the gap (index hospitalization).

The Time 2 variable allows the model to have different slope in period 2 than in period 1. The Time 4 variable allows the model to have a different slope in period 4 than in period 3.

The time variables for periods 1 and 3 are interpreted as the average weekly change in the odds of a fall injury within each of those respective periods. For instance, an OR of 1.10 in period 1 indicates that the odds of a fall injury in week 2 of period 1 (or 3) are 110% those of the odds in week of period 1 (or 3). However, the ORs in periods 2 and 4 indicate the difference in the odds of a fall injury compared to those in periods 1 and 3, respectively. For instance, if the OR for periods 1 and 2 are both >1, that indicates that the slope of period 2 is greater than the slope of period 1, i.e., that the average odds of a fall injury for each week spent in periods 1 and 2 increase, but the odds of injury increase more in period 2 than in period 1.

It is possible, however, to use the slopes of periods 1 and 2, and then of periods 3 and 4, to compute ORs for period 2 and 4 that do not reflect the relative change in odds of an injury compared to periods 1 and 3, respectively; instead, for periods 2 and 4, we can compute the average change in the odds of a fall injury for those periods. In other words, we can produce ORs that are interpreted in the same ways for periods 2 and 4 that we interpret them for periods 1 and 3.[1]

The odds ratios and marginal effects for piecewise logistic regression models estimated using each of the two fall injury algorithms (conservative and aggressive approaches) are shown below.

[1] Ryan, S. E., & Porth, L. S. (2007). *A Tutorial on the Piecewise Regression Approach Applied to Bedload Transport Data*. Retrieved from https://www.fs.fed.us/rm/pubs/rmrs_gtr189.pdf

© 2020 Hoffman GJ et al. *JAMA Network Open.*

eTable 4. Odds Ratios and Marginal Effects Using Conservative Approach

	Odds Ratio	95% CI		P values	Marginal effects	95% CI	P values
Baseline	1.02	1.00	1.03	0.000	0.000	0.000, 0.000	0.001
Before hospitalization	1.37	1.26	1.49	0.002	0.002	0.001, 0.002	0.000
After hospitalization	0.87	0.81	0.94	-0.002	-0.002	-0.002, -0.001	0.000
Follow-up	1.00	0.99	1.01	0.000	0.000	0.000, 0.000	0.986

© 2020 Hoffman GJ et al. *JAMA Network Open.*

eTable 5. Odds Ratios and Marginal Effects Using Aggressive Approach

	Odds Ratio	95% CI		P values	Marginal effects	95% CI	P values
Baseline	1.02	1.00	1.03	0.013	0.000	0.000, 0.000	0.001
Before hospitalization	1.40	1.29	1.52	0.000	0.003	0.002, 0.003	0.000
After hospitalization	0.80	0.75	0.86	0.000	-0.002	-0.002, -0.001	0.000
Follow-up	1.00	0.99	1.01	0.699	0.000	0.000, 0.000	0.781

© 2020 Hoffman GJ et al. *JAMA Network Open.*

eTable 6. Fall Injury Rates (per 1,000 Patient Days) of Older (≥65) Individuals, Relative to Hospitalization, 2008-2014 (*n*=10,106)

	50 weeks			Following 4 weeks*			Preceding 4 weeks*		
**Approach**	No. fall injuries	Total Days	Rate	No. fall injuries	Total Days	Rate	No. fall injuries	Total Days	Rate
**Conservative**	2,354	3,109,213	0.757	348	276,822	1.257	407	278,143	1.463
**Aggressive**	2,386	3,100,927	0.769	412	275,372	1.496	430	277,883	1,547

* Relative to hospitalization

© 2020 Hoffman GJ et al. *JAMA Network Open.*

eTable 7. Marginal Effects (MEs), and 95% Confidence Intervals (CI) for a Fall Injury for Older (≥65) Adults with a Pre-Hospital (Month Before Hospitalization) Fall During Four Time Periods Before and After a Hospitalization by Injured Body Part (*n*=433)

	**Head (*n*=89)**		**Neck/Trunk (*n*=99)**		**Upper Body (*n*=99)**		**Lower Body (n=148)**	
	ME (pp+)	95% CI	ME (pp+)	95% CI	ME (pp+)	95% CI	ME (pp+)	95% CI
Baseline	0.38	0.22, 0.54	0.46	0.29, 0.63	0.50	0.29, 0.71	0.48	0.31, 0.66
Before hospitalization	2.82	2.19, 3.45	2.27	1.74, 2.79	2.93	2.29, 3.57	2.68	2.08, 3.29
After hospitalization	-0.90	-1.75, -0.06	-0.47	-1.36, 0.43	-1.55	-2.36, -0.75	-0.81	-1.48, -0.04
Follow-up	-0.06	-0.18, 0.07	-0.06	-0.18, 0.06	0.02	-0.08, 0.13	-0.13	-0.24, -0.01

* $p < 0.05$. + pp = percentage point

Note: 'Baseline' refers to 6 to 1 month before hospitalization; 'Before hospitalization' refers to less than 1 month before hospitalization; 'After hospitalization' refers to from discharge to 1 month following discharge; 'Follow-up' refers to 1 to 6 months following discharge. Estimates were obtained from a piecewise logistic regression model that included dummy variables for each of the 4 time periods of interest (e.g., baseline, before hospitalization periods), using 2008-2014 data for Medicare beneficiaries with linked Health and Retirement Study data.

© 2020 Hoffman GJ et al. *JAMA Network Open.*

eTable 8. Sensitivity Analyses Indicating Marginal Effects and 95% Confidence Intervals (CI) for a Fall Injury for Older (≥65) Adults during Four Time Periods Before and After a Hospitalization

	**Shorter Period between Survey and Study Period** [a]	**Dropping First Hospitalization** [b]	**180-day Lookback for Defining Fall Injury** [c]
**Baseline**	0.02* (0.00, 0.02)	0.01* (0.01, 0.02)	0.01* (0.00, 0.01)
**Before hospitalization**	0.27* (0.19, 0.33)	0.19* (0.14, 0.25)	0.17* (0.13, 0.21)
**After hospitalization**	-0.18* (-0.22, -0.09)	-0.17* (-0.23, -0.12)	-0.14* (-0.19, -0.10)
**Follow-up**	0.00 (-0.01, 0.01)	0.00 (-0.01, 0.00)	0.00 (0.00, 0.01)

* $p < 0.05$. + pp = percentage point

[a] Only included observations for individuals for whom the time between the date of their HRS survey and start of the study period (6 months prior to the anchor hospitalization) was ≤365 days.

[b] Dropped first hospitalizations for individuals with more than one hospitalization.

[c] Fall injury diagnoses were included in a single fall injury episode if they occurred within 180 days of one another (i.e., if an ankle fracture was treated in the emergency department on April 2, 2013 and a foot injury was treated in a physician's office on September 12, 2013, both diagnoses would be considered to be part of a single fall injury episode that occurred on April 2, 2013.

© 2020 Hoffman GJ et al. *JAMA Network Open.*
